# *Drosophila* SPF45: A Bifunctional Protein with Roles in Both Splicing and DNA Repair

**DOI:** 10.1371/journal.pgen.0020178

**Published:** 2006-12-08

**Authors:** Ahmad Sami Chaouki, Helen K Salz

**Affiliations:** Department of Genetics, School of Medicine, Case Western Reserve University, Cleveland, Ohio, United States of America; Stowers Institute for Medical Research, United States of America

## Abstract

The sequence of the SPF45 protein is significantly conserved, yet functional studies have identified it as a splicing factor in animal cells and as a DNA-repair protein in plants. Using a combined genetic and biochemical approach to investigate this apparent functional discrepancy, we unify and validate both of these studies by demonstrating that the Drosophila melanogaster protein is bifunctional, with independent functions in DNA repair and splicing. We find that SPF45 associates with the U2 snRNP and that mutations that remove the C-terminal end of the protein disrupt this interaction. Although animals carrying this mutation are viable, they are nevertheless compromised in their ability to regulate *Sex-lethal* splicing, demonstrating that *Sex-lethal* is an important physiological target of SPF45. Furthermore, these mutant animals exhibit phenotypes diagnostic of difficulties in recovering from exogenously induced DNA damage. The conclusion that SPF45 functions in the DNA-repair pathway is strengthened by finding both genetic and physical interactions between SPF45 and RAD201, a previously uncharacterized member of the RecA/Rad51 protein family. Together with our finding that the fly SPF45 protein increases the survival rate of mutagen-treated bacteria lacking the RecG helicase, these studies provide the tantalizing suggestion that SPF45 has an ancient and evolutionarily conserved role in DNA repair.

## Synopsis

Assigning function to a protein relies on information about similar proteins in different species, and is based on the view that conservation of sequence generally parallels conservation of function. In this article, Chaouki and Salz focus on SPF45, a protein that, at first glance, appears to break this rule. Although the sequence of SPF45 is highly conserved, in animals cells SPF45 functions as a splicing factor, but in plant cells it functions as a DNA repair protein. This functional discrepancy is resolved here through the demonstration that, in *D. melanogaster,* SPF45 is a bifunctional protein with independent functions in DNA repair and splicing. Support for this conclusion includes the observation that mutant animals lacking SPF45 function display defects in both splicing and DNA repair. In addition, the authors show that SPF45 associates with two distinct groups of proteins; those that participate in RNA splicing and those that participate in DNA repair. The finding that the D. melanogaster protein is bifunctional suggests that the human protein may also have more than one function. This has important clinical implications because elevated SPF45 levels have been correlated with resistance to chemotherapy.

## Introduction

Assigning function to a newly discovered protein relies heavily on our understanding of similar proteins in other species. While for the most part the convergence of information from multiple systems points to a single function, occasionally this information leads to disparate cellular roles. Without additional functional studies, it is difficult to establish whether the conservation of sequence through evolution reflects mechanistic conservation, implying that the protein has more than one purpose, or, alternatively, that there has been significant divergence of function over time. One particularly interesting example is SPF45, because although its protein sequence shows significant conservation between plants and animals (Figure 1), its function is not thought to be conserved. In animals, SPF45 is identified as a splicing protein, while in plants it is labeled as a DNA-repair protein. The similarity in sequence between humans, flies, and plants is especially striking within the G-patch domain and the RNA-recognition motif (RRM). These two domains are often, but not exclusively, found in proteins that function in RNA processing. Indeed, the specificities of these domains appear to be quite diverse, with RNA-, single-stranded–DNA-, and protein-binding capabilities [1–7]. Thus, while the SPF45 protein contains domains that are considered canonical hallmarks of an RNA-binding protein, the actual substrate diversity of these domains is consistent with the possibility that SPF45 has two independent functions.

**Figure 1:**
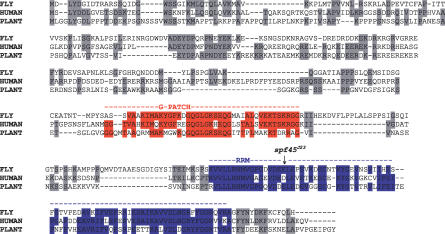
D. melanogaster SPF45 Protein Is Conserved Alignment of SPF45 proteins from D. melanogaster and humans and the DRT111 protein from A. thaliana. Identical residues are shaded. The residues that comprise the G-patch domain are shaded in red, and the residues that comprise the RRM-like motif are shaded in blue. An arrow indicates the position of the *P-element* insertion in *spf45^J23^*.

The classification of the Arabidopsis thaliana SPF45 protein (originally called DRT111) as a DNA-repair protein is based on its ability to rescue the mutagen-sensitive phenotype of the *Escherichia coli recG* mutant [8]. RecG, a branch-migration helicase for which there is no known eukaryotic equivalent, is required to process the Holliday junctions formed after DNA damage [9]. Although the mechanism by which expression of the nonhomologous SPF45 gene can functionally complement *recG* is not known, this ill-defined function is likely to have appeared very early in eukaryotic evolution, because the more distantly related SPF45 counterpart from the protozoan parasite Toxoplasma gondii is also capable of restoring function to the same E. coli mutant strain [10]. Interestingly, while proteins with homology to SPF45 are present in several different protozoans, SPF45 counterparts are not identifiable in the genomes of the budding yeast *Saccharomyces cerevisiae* or the fission yeast *Schizosaccharomyces pombe,* raising questions about its evolutionary history.

There have been no studies to date linking the metazoan SPF45 protein to DNA repair, but there is a substantial body of evidence that suggests a role in RNA splicing. The link between SPF45 and splicing was first made when the protein was identified as a component of the human spliceosome by mass spectrometry [11]. Spliceosomes are macromolecular protein/RNA machines that assemble from a set of smaller complexes and factors in a step-wise fashion [12,13]. Many of these subcomplexes have been purified and a detailed description of the proteomic composition of the spliceosome as it assembles is now available [14–17]. In these studies, SPF45 was identified as one of a small group of proteins that associates with the 17S U2 snRNP early in spliceosome assembly [17], but then dissociates from the complex as the spliceosome matures and becomes catalytically active [15,16]. Consistent with its identity as a U2 snRNP protein, functional studies carried out in HeLa cell extracts have shown that SPF45 plays a role in 3′ splice–site utilization [18].

SPF45′s function in splicing is likely to be conserved from humans to flies because studies in D. melanogaster tissue-culture cells have suggested that SPF45 plays a role in controlling the alternative splicing of the *Sex-lethal (Sxl)* pre-mRNA [18]. In *D. melanogaster,* the sex-specific activity of the developmental switch gene *Sxl* is controlled by splicing [19,20]. In males, where *Sxl* must remain off, no full-length protein is produced because the third exon containing translational stop codons is included in the mRNA. In females, where *Sxl* activity is essential, the third exon is skipped, resulting in protein-producing mRNAs. There is considerable genetic and biochemical evidence supporting a model in which *Sxl* negatively regulates splicing of its own pre-mRNA by interrupting the process of spliceosome assembly through interactions with the U1 snRNP on the 5′ side of the male exon and the U2AF heterodimer on the 3′ side of the male exon [21]. Although not yet confirmed by genetic studies, SPF45′s role in this process appears to be significant, as depletion of SPF45 by RNAi has a negative impact on the efficiency of male-exon skipping in tissue-culture cells [18]. Furthermore, this effect is likely to be direct, as in vitro experiments have shown that SPF45 is capable of both interacting with the SXL protein and binding to the 3′ splice site of the *Sxl* male exon [18].

In this study, we investigate the functional repertoire of D. melanogaster SPF45. Our studies unify and validate previous studies of this protein, as we have discovered that it functions in both splicing and DNA repair. Our genetic studies are complemented by a biochemical analysis, which demonstrates that SPF45 can physically interact with core components of the spliceosome as well as Rad201*,* a previously uncharacterized member of the RecA/Rad51 protein family. Together, these results provide compelling evidence that SPF45 is a bifunctional protein with independent functions in DNA repair and splicing.

## Results/Discussion

### The D. melanogaster Genome Encodes a Single SPF45 Homolog That Is Embedded within Heterochromatin

The D. melanogaster genome encodes a single candidate gene, CG17540, that shares significant homology with the human SPF45 protein and the A. thaliana DRT111 protein (Figures 1 and 2A). Based on this sequence conservation and the functional studies described below, we have named the D. melanogaster CG17540 gene *spf45*.

**Figure 2:**
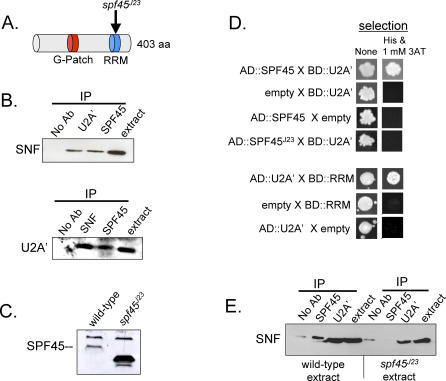
Association between SPF45 and the U2 nsRNP Is Mediated by a Direct Interaction between the C-Terminal End of SPF45 and U2A′ (A) Diagram of the 403–amino-acid SPF45 protein. An arrow indicates the position of the *P-element* insertion in *spf45^J23^.* (B) Western blots of U2A′, SPF45, and SNF immunoprecipitations (IP) from nuclear extracts showing that SPF45 can be co-immunoprecipitated with SNF and U2A′. Previous studies have shown that SNF and U2A′ directly interact [26] and are included here as positive controls. (C) Western blot of extracts made from wild-type and *spf45* mutant animals probed with a polyclonal antibody against SPF45 shows that the expected size band is replaced by an ∼37 kD band in *spf45^J23^*. In extracts made from adults (but not in nuclear extracts made from embryos), the SPF45 antibody picks up an additional higher molecular weight cross-reacting band of unknown origin, which can serve as a loading control. (D) Yeast two-hybrid interactions showing that SPF45 interacts with U2A′, and that this interaction is mediated by the RRM domain. Positive interactions were tested by assaying the ability of the transformed yeast to grow on selective media after 3 days. (E) Western blots of U2A′ and SPF45 immunoprecipitations (IP) from wild-type and mutant lysates showing that the SPF45^J23^ does not co-immnoprecipitate with SNF in mutant extracts, whereas the control splicing proteins, SNF and U2A′, are co-precipitated.

Interestingly, the *spf45* gene is located at the edge of the annotated genome within the heterochromatic region h35 on the left arm of Chromosome 2 [22]. *spf45* is part of a small gene cluster that has only recently moved from a euchromatic location to heterochromatin [23]. This transition, which occurred about 20 million years ago in the drosophilid lineage, was not accompanied by any major changes in promoter structure, indicating that *spf45*, and its neighbors, are functional single-copy genes that happen to be embedded within heterochromatin [23].

### The D. melanogaster SPF45 Protein Associates with the Splicing Machinery

The assignment of a role in splicing to SPF45 was initially based on its identification as a component of the highly purified human 17S U2 snRNP particle, by mass spectrometry [17]. To establish that D. melanogaster SPF45 associates with the U2 snRNP, we generated an antibody for co-immunoprecipitation assays. As shown in Figure 2B, we find that antibodies directed against SPF45 can in fact co-immunoprecipitate the two core U2 snRNP-specific proteins, SNF and U2A′ [24–26]. Furthermore, we can detect an interaction between SPF45 and U2A′ in a yeast two-hybrid assay (Figure 2D), indicating that this interaction links the D. melanogaster SPF45 protein to the U2 snRNP. From these data, we conclude that in D. melanogaster, as in mammalian cells, SPF45 associates with the U2 snRNP.

### A Deletion That Removes the C-Terminus of SPF45 Fails to Associate with the U2 snRNP, but Is Nevertheless Viable and Fertile

To identify mutations in the *spf45* gene, we searched the D. melanogaster databases for *P-element* insertions located in or near *spf45* and identified a viable and fertile *P-element* insertion, called KG2503, which contains an insertion located within the coding sequence of *spf45* [27]. Using this *P*-insertion as a starting point, we screened for *P element*–excision events (see Materials and Methods), and identified two derivatives: *spf45^J23^* and *Df(2Lh)D1*.

Using a combination of genetic and molecular approaches, we determined that *Df(2Lh)D1* is a deletion that removes the entire *spf45* coding sequence as well as the adjacent genes, *concertina* and *Chitinase 3* (unpublished data). Thus, we estimate that at a minimum *Df(2Lh)D1* deletes 80 kb of DNA [22]. *Df(2Lh)D1* also fails to complement *l(2)40Fd*, *l(2)40Ff,* and *l(2)40Fe,* three previously identified lethal mutations that have not yet been linked to specific coding sequences [28]. To saturate the region for lethal alleles, we carried out an additional screen for lethal mutations that fail to complement *Df(2Lh)D1.* In our screen of ∼9,000 EMS-treated chromosomes, we identified seven additional alleles of *l(2)40Fd* and one additional allele of *l(2)40Ff,* but no novel lethal-complementation groups. Thus, we conclude that the genomic region deleted in *Df(2Lh)D1* contains three genes that mutate to lethality. None of the three complementation groups, however, is likely to correspond to SPF45, because 1) they do not contain mutations within the SPF45-coding sequence, 2) the lethal phenotypes are not rescued by *P{spf45+}*, a transgene carrying 3.1 kb of genomic DNA that contains only the *spf45* transcription unit, and 3) each lethal allele complements both the mutagen sensitivity and *Sxl* splicing defective phenotypes of *spf45^J23^* described below (unpublished data). Together, these data lead us to the tentative conclusion that loss of *spf45* function does not result in lethality.

To continue investigating the function of SPF45, we focused our analysis on *spf45^J23^,* a viable and fertile allele that retains 1 kb of the *P-element* insertion located within the coding sequence (Figure 2A). Based on its sequence, *spf45^J23^* is predicted to encode a truncated protein of 37 kD, containing the first 312 amino acids of SPF45 followed by an additional three amino acids of novel sequence. As predicted, we find that on Western blots the wild-type protein migrates at about 45 kD and the SPF45^J23^ migrates at about 37 kD in extracts made from adult flies (Figure 2C). Unlike the wild-type protein, the truncated SPF45^J23^ protein could only be detected in adult extracts and not in extracts made from embryos (unpublished data).

The truncated SPF45^J23^ protein is missing the majority of its conserved RRM-like motif, a domain that can mediate protein–protein interactions [1]. We therefore tested whether this motif can mediate the interaction with U2A′ in the yeast two-hybrid assay. As illustrated in Figure 2D, we find that expression of the RRM-like motif alone (amino acids 304–403) is sufficient to mediate the interaction with U2A′ and that a partial deletion of this region (identical to the endogenous SPF45^J23^ protein) abolishes the interaction. To investigate whether the association between SPF45^J23^ and the U2 snRNP is compromised in vivo, we asked whether the endogenous wild-type or mutant SPF45 protein could associate with the core U2 snRNP protein, SNF, in extracts made from wild-type and *spf45^J23^* mutant embryos. In these co-immunoprecipitation experiments, we failed to detect an interaction between SPF45^J23^ and SNF (Figure 2E). While it is possible that the relatively low abundance of the mutant protein in embryonic extracts could explain why an interaction is not detected, given the sensitivity of the assay, we think it more likely that without an intact RRM-like motif, SPF45^J23^ does not form a stable association with the U2 snRNP.

Our genetic studies, described in more detail below, also suggest that this truncated protein is likely to be nonfunctional, because in all cases the mutant phenotype of *spf45^J23^/Df(2Lh)D1* was no more severe than the phenotype of *spf45^J23^* homozygous mutant animals. Together, these studies lead us to conclude that *spf45^J23^* eliminates *spf45* function and that the loss of *spf45* function does not cause lethality. Given that the U2 snRNP is integral to the splicing reaction, our finding that *spf45^J23^* mutant animals are viable and fertile argues that SPF45 is not essential for the role of U2 snRNP in general splicing. While it remains possible that disruption of the functions attributed to SPF45 from in vitro splicing assays and cell-culture studies are simply not important in the animal, we suggest instead that the impact of depleting SPF45 on its physiologically important targets does not lead to lethality. Support for this view comes from our more detailed analysis of the *spf45* mutant phenotype, described below.

### SPF45 Influences the Efficiency of *Sxl* Male Exon Skipping

Previous studies in D. melanogaster tissue-culture cells have established that a reduction in *spf45* levels has a dramatic effect on the efficiency of *Sxl* male exon skipping [18]. We therefore anticipated that *spf45* mutations might have a significant effect on the efficiency of *Sxl* male exon skipping in a genetically sensitized background. We have previously shown that embryos heterozygous for the normally recessive null allele of *Sxl* (*Sxl^f1^/+*) provide a sensitized background in which to assay for splicing defects [21,29]. Our studies have shown that *Sxl^f1^/+* heterozygous embryos are particularly sensitive to the supply of specific splicing proteins deposited into the egg by the mother. If maternally provided SPF45 protein is important for *Sxl* male exon skipping, then we would expect a reduction in exon skipping and protein production in the *Sxl^f1^/+* daughters of *spf45^J23^* homozygous mutant mothers. Because we expect that a major reduction in *Sxl* male exon skipping will result in female lethality, we first assayed the survival rate of *Sxl^f1^/+* daughters of *spf45^J23^* homozygous mutant mothers compared with controls. As expected, we found a significant impact on survival, with only 4% of the expected *Sxl^f1^/+* daughters surviving (Figure 3A). Restoration of female viability by the genomic rescue construct, *P{spf45+}*, indicates that this female-lethal synergistic interaction is due to the loss of *spf45* function.

**Figure 3:**
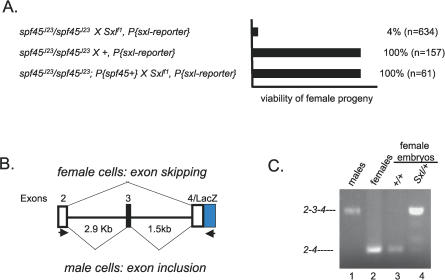
*Sxl* Splicing Is Compromised in a *spf45* Loss-of-Function Background (A) Synergistic genetic interactions between *Sxl* and *spf45^J23^* leads to female lethality. In these assays females of the indicated genotype were mated to either *Sxl^f1^/Y* males or *Sxl^+^/Y* control males and the resulting progeny scored. On the assumption that an equal number of male and female progeny should be generated from each cross, the percent female viability was calculated by comparing the number of females recovered with the number of males recovered. (B) Diagram of the *Sxl* reporter construct that mirrors native *Sxl* splicing in all tissues tested. The arrows below the construct indicate the position of the PCR pairs used for RT–PCR. (C) Synergistic lethal interactions between *Sxl* and *spf45^J23^* are correlated with *Sxl* splicing defects. Splicing was assayed by an RT–PCR-based assay using RNA isolated from a pool of embryos in which only the female embryos carry the reporter construct (lanes 3 and 4). In lane 4, this pool of embryos was collected from *spf45^J23^* homozygous females crossed to males carrying an X chromosome that carries both *Sxl^f1^* and a copy of the *Sxl* reporter construct. Controls include splicing of the reporter construct in adult males (lane 1), splicing of the reporter construct in adult females (lane 2), and *Sxl^+^* embryos collected from *spf45^J23^* homozygous mothers (lane 3).

To determine whether this synergistic female lethality is correlated with an increase in *Sxl* male exon inclusion, we assayed *Sxl* splicing using an X-linked reporter construct that is known from previous studies to faithfully reproduce the endogenous splicing pattern (Figure 3B) [21,30]. Linkage to the X chromosome allows us to produce a population of embryos in which females carry the reporter construct and males do not. For example, when RNA is isolated from embryos collected from *spf45^J23^* homozygous mutant females crossed to males carrying the X-linked reporter construct, only the female *Sxl*-reporter spliced product is detectable (Figure 3C, lane 3). In sharp contrast to the above results, when embryos are collected from the same *spf45^J23^* mothers crossed to males carrying the X-linked *Sxl^f1^* null allele and the reporter construct, the vast majority of the spliced products that are detected contain the male-specific exon (Figure 3C, lane 4). The female lethality and switch in splicing pattern observed in this sensitized background indicate that *spf45* contributes to the efficiency of *Sxl* male exon skipping.

Based on these studies, we conclude that *Sxl* is a physiologically important target of SPF45, in agreement with the conclusions drawn from RNA-interference knock-down experiments in tissue-culture cells [18]. By carrying out this analysis in *D. melanogaster,* we were able to establish that depletion of *spf45* in an otherwise wild-type background does not have an impact on survival of the animal, and only becomes essential when the pathway leading to male exon skipping is otherwise compromised.

### SPF45 Is Required for DNA Repair

Having provided definitive evidence that D. melanogaster SPF45 plays a role in pre-mRNA splicing, we wanted to test whether the other function ascribed to this protein is also conserved. A role for SPF45 in DNA repair was suggested by the observation that expression of the A. thaliana SPF45 protein in E. coli partially rescues the mutagen sensitivity of the *recG* mutant strain [8]. The E. coli RecG protein is a helicase that functions in recovery from DNA damage by resolving the Holliday-junction structures generated during the repair process. In studies that parallel those of Pang et al. [8], we find that expression of the D. melanogaster SPF45 protein in *E. coli recG* mutants confers a moderate, but statistically significant, resistance to the DNA alkylating agent, MMS (Figure 4A). Furthermore, we found expression of the mutant SPF45^J23^ protein in E. coli fails to provide any protection, indicating that the SPF45^J23^ mutant protein is nonfunctional in this assay. Thus, in a manner analogous to the plant protein, D. melanogaster SPF45 either functionally replaces or somehow allows for bypass of the function provided by the RecG helicase.

**Figure 4:**
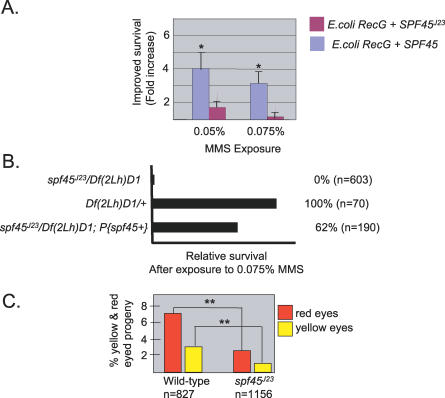
SPF45 Function Is Required to Repair DNA Damage (A) Ectopic expression of SPF45 improves the survival rate of *E. coli recG* mutants after MMS exposure. The survival was expressed as the average-fold increase in survival over the control *E. coli recG* mutants. Error bars are standard deviations calculated from five independent experiments. An unpaired *t* test was performed to assess whether there is a statistically significant difference between survival rates of an *E. coli recG* strain expressing a D. melanogaster protein (either SPF45^J23^ or SPF45^+^) and the survival rate of the same *E. coli recG* strain expressing an empty GST vector*.* Asterisks denote differences significant at the 95% level (*p* < 0.04). (B) *spf45* animals have an MMS-sensitive mutant phenotype. Survival was assessed by comparing the number of mutant animals that survived to adulthood with the number of control siblings (*spf45^J23^/CyO* or *Df(2Lh)D1/CyO*) recovered from the same cross. (C) Damage after *P-element* excision is not repaired efficiently in an *spf45* mutant background. Eye colors of female progeny that inherited the repaired *P{w^a^}* were scored. The total number of females scored is indicated below the relevant genotype. A chi square test was performed to assess whether there is a significant difference between the frequency of repair events. Asterisks denote differences significant at the 99.9% level (*p* < 0.0005).

The finding that SPF45 is capable of functional rescue in a heterologous system begs the question of relevance. To ascertain whether SPF45 is required for DNA repair in *D. melanogaster,* we tested whether the loss of SPF45 function rendered the flies sensitive to DNA damage induced by feeding MMS to mutant larvae. Previous studies have shown that while wild-type larvae can cope with the damage induced by low levels of MMS, animals mutant for genes required for DNA repair fail to recover and die [31]. As illustrated in Figure 4B, we found that in contrast to control animals, *spf45^J23^*-mutant animals did not survive exposure to MMS. Importantly, the MMS-sensitive phenotype of these animals is rescued by the genomic rescue construct, *P{spf45+},* indicating that the mutagen sensitivity is in fact due to the loss of *spf45* function. Based on these observations, we conclude that *spf45* function is required to promote DNA repair.

To further establish this point, we tested whether *spf45* is required to repair the damage created during transposition of a *P-element* transposable element. Since the failure to repair damaged DNA can lead to cell death, we expect the loss of *spf45* function to adversely affect the recovery of animals that have successfully repaired the break caused by *P-element* transposition. We tested this prediction using a recently described assay in which the fate of a single X-linked *P{w^a^}* transposable element can be followed by changes in eye color [32]. In this assay, the recovery of red-eyed female progeny is diagnostic of successful DNA repair by a homologous recombination mechanism called synthesis-dependent strand-annealing. In our wild-type background, 7% (*n* = 827) of the progeny have red eyes. Progeny with yellow eyes, on the other hand, indicate the use of other repair pathways, including nonhomologous end joining or incomplete synthesis-dependent strand-annealing repair. In our wild-type background, 3% of the female progeny (*n* = 827) had yellow eyes. In contrast to our control studies, only 2.4% of the female progeny from *spf45* mutants had red eyes and 1.2% had yellow eyes (*n* = 1156). Thus, in the *spf45* mutant, the overall rate of recoverable events is reduced from 10% to 3.6%, suggesting that the defective protein abrogates effective repair of the DNA breaks induced during transposition.

Programmed DNA breaks and their subsequent repair are a necessary part of meiotic recombination, thus raising the question of whether *spf45* also functions in the resolution of meiotic recombination intermediates. To answer this question, we measured the frequency of X-chromosome nondisjunction, because recombination is part of the mechanism that ensures the accurate segregation of homologous chromosomes, and mutations that interfere with recombination also display high levels of chromosome nondisjunction. In this assay, however, *spf45^J23^*-mutant animals have an ∼0.3% level of nondisjunction, which is no higher than the level observed in wild-type animals (unpublished data). Based on these data, we conclude that SPF45 does not play a role in repairing the breaks generated during meiotic recombination.

Based on these studies, we conclude that D. melanogaster SPF45 plays a significant role in the pathway leading to repair of exogenously induced DNA damage, without being essential for repair of programmed DNA damage.

### Genetic and Physical Interactions between SPF45 and RAD201, a RecA/Rad51 Family Member

Given that mutations in *spf45* have a classical mutagen-sensitive phenotype, we wondered whether additional *spf45* mutant alleles might be found in collections of viable, mutagen-sensitive mutations that map to the second chromosome [31]. We therefore carried out complementation tests with representative alleles of 18 different second-chromosome genes with MMS-sensitive mutant phenotypes. Mutations in only one gene, *rad201,* failed to complement the mutagen-sensitive phenotype of *spf45^J23^* (unpublished data). Upon further analysis, we found nothing unusual about this interaction; the failure to complement is not dependent on which allelic combinations are used, and the addition of the *P{spf45+}* genomic construct fully rescues the mutagen-sensitive phenotype (Figure 5A). Nevertheless, it seemed unlikely that *rad201* was a bona fide mutant allele of *spf45* for three reasons. First, previous studies had mapped *rad201* to the right arm of Chromosome 2 (see http://www.flybase.org), whereas SPF45 maps to the left arm. Second, sequencing of the *rad201^1^* and *rad201^ZII670^* mutant strains did not identify any mutations in the *spf45* coding sequence (unpublished data). Third, *rad201* mutations, by themselves, or in combination with *spf45^J23^*, have no impact on female viability, even in a sensitized background, suggesting that *rad201* does not contribute to the efficiency of *Sxl* male exon skipping (unpublished data).

**Figure 5:**
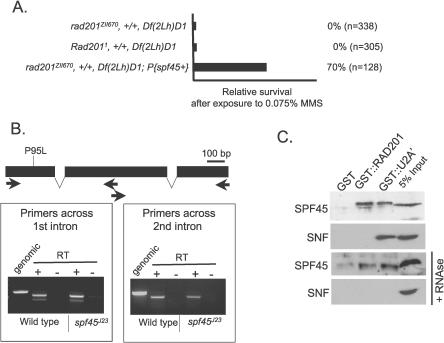
Genetic and Physical Interactions between SPF45 and RAD201, a Member of the RecA/Rad51 Protein Family (A) *spf45^J23^* does not complement the MMS mutant phenotype of *rad201*. Relative survival to adulthood of the double heterozygous mutant animals was assessed by comparing the number of experimental animals with the number of sibling controls. (B) *rad201* splicing in wild-type and *spf45^J23^* mutants. Genomic organization of *rad201* (CG2412). The position of the C-to-T transition leading to a proline-to-leucine change at position 95 in the *rad201^1^* open reading frame is indicated. The *rad201* gene encodes a single transcript. RT–PCR analysis using primers that span the first and second introns demonstrates that splicing is unaffected in *spf45^J23^* mutants. (C) RAD201 interacts with SPF45 in whole-cell extracts in an RNA-independent manner. The ability of RAD201 to associate with SPF45 in whole-cell extracts was tested by GST pull-down assays followed by Western blotting. GST::RAD201, GST::U2A′, or GST alone, bound to glutathione sepharose beads, were incubated with extracts made from wild-type or *spf45^J23^* embryos, followed by Western blotting using an antibody directed against SPF45 or SNF. The lane marked 5% input is a control in which the amount of extract corresponds to ∼5% of the material applied to the glutathione affinity beads. The RNase sensitivity of these interactions was tested by pretreating the extracts with a combination of RNase A and RNase T1.

We conclude, therefore, that we have uncovered an example of intergenic or second-site noncomplementation. This interaction is quite specific; mutations in other splicing factors, such as *snf, U2A*′, *U2AF38,* and *U1-70K*, all complement the mutagen-sensitive phenotype of *rad201^1^* (unpublished data). Finding that mutations at two different loci fail to complement each other in an allele-independent manner, as is the case here, raises the possibility that both genes have functions that impact on a common pathway, i.e., DNA repair.

*rad201* maps close to CG2412, a member of the RecA/Rad51 protein family. The possibility that RAD201 might belong to a RAD51/RECA protein family is particularly intriguing, because all members of this protein family have clear, albeit distinct, roles in repairing DNA damage, and others have in fact suggested that *rad201* might be encoded by CG2412 [33,34], even though *rad201* was initially reported to correspond to the adjacent gene, CG8016 (http://www.flybase.org). We therefore sequenced the open reading frames of both genes in *rad201^1^* homozygotes. Whereas we did not detect any mutation causing base-pair changes in CG8016, we did identify a single nucleotide substitution in CG2412 that resulted in the conversion of proline to leucine at position 95 (CCC→CTC, Figure 5B), a region of the protein that is highly conserved between D. melanogaster and Drosophila pseudoobscura. Although no mutations were identified in *rad201^ZII670^*, we are confident that *rad201* corresponds to CG2412, because, as we show below, the protein encoded by CG2412 interacts with SPF45. We shall therefore refer to CG2412 as *rad201*.

What is the molecular basis of intergenic noncomplementation? Given that SPF45 is a component of the spliceosome, we first considered the possibility that mutations in *spf45* might have a detrimental effect on *rad201* gene expression by reducing the efficiency of splicing for one or both introns. To investigate this possibility, we analyzed the RNA products of the *rad201* gene in *spf45^J23^* mutants using RT–PCR followed by sequence analysis. As illustrated in Figure 5B, we detected no differences in *rad201* splicing between wild-type and *spf45* mutant animals. The failure of *spf45* to complement *rad201* mutants therefore is unlikely to arise from a reduction in *rad201* gene expression caused by a splicing defect.

Other examples of intergenic noncomplementation have been described between mutations in genes whose products function within the same multiprotein complex (e.g., [35]). To explore this possibility, we asked whether the RAD201 and SPF45 proteins are capable of forming a complex in whole-cell extracts. Since antibodies against the fly RAD201 protein are not available, complex formation was assayed by pull-down experiments in which a GST::RAD201 fusion protein was expressed in bacteria, bound to glutathione sepharose beads, and incubated with protein extracts made from embryos. The presence or absence of SPF45 in the complexes formed on the beads was assayed by Western blot analysis (Figure 5C). In control studies, we used a GST::U2A′ fusion protein, since it is known to form a complex with SPF45. Using this assay, we found that GST::RAD201, but not GST alone, was capable of selecting SPF45 out of extracts as efficiently as its known partner protein, GST::U2A′. Furthermore, even though GST::U2A′ can select SNF from extracts, the formation of a GST::RAD201/SNF complex is not detectable, suggesting that RAD201 does not interact with SPF45 in the context of the spliceosome. To further establish this point, we carried out these experiments in the presence of RNase to digest the snRNAs present in the extract. This treatment disrupts the SNF-U2A′ interaction, but RAD201 and SPF45 were found to interact in an RNA-independent manner. Thus, it is clear that there are both genetic and physical interactions between SPF45 and RAD201. Collectively, these findings further support the conclusion that SPF45 is a bifunctional protein.

### Concluding Remarks

The repair of damaged DNA and splicing of newly transcribed RNA both require the cooperative functions of many different proteins for the selection and positioning of the nucleic-acid substrate for enzymatic processing. Thus, one might imagine that there will be proteins that function in both pathways, and we would suggest that SPF45 is such a protein. While studies directly investigating whether the vertebrate SPF45 protein also functions in DNA repair have not been carried out, recent gain-of-function and loss-of-function studies in human cancer–cell lines are suggestive. In these studies, a reduction in SPF45 expression in A2780 cells, an ovarian carcinoma cell line, resulted in chemotherapeutic drug sensitivity [36]. Conversely, ectopic overexpression of SPF45 in both A2780 cells and HeLa cells rendered the cells resistant to several different anticancer drugs [36,37]. Based on the observation that a number of solid tumors appear to have excessively high levels of SPF45, the authors suggest that increased accumulation of SPF45 plays a role in the clinical resistance to chemotherapy seen in some solid tumors [36,37]. Indeed, expression screening of a drug-resistant variant of the mouse mammary tumor–cell line EMT6 led to the initial isolation of SPF45 by these authors [37]. Our finding that SPF45 is a bifunctional protein has important implications for future studies directed towards understanding the mechanism by which elevated SPF45 levels contribute to the chemo-resistant phenotype of cancer cell lines.

## Materials and Methods

### Antibodies, co-immunoprecipitations, and Western blots.

The antibody against D. melanogaster SPF45 was raised in guinea pig (Covance Research Products, http://www.crpinc.com) against full-length HIS-tagged SPF45 purified from bacteria. The other antibodies used in this study, anti-SNF [38] and anti-U2A′ [26], have been described previously. To make protein extracts from adults, D. melanogaster of the appropriate genotype were homogenized in NET buffer (150 mM NaCl, 5 mM EDTA, 50 mM Tris, pH 7.5) supplemented with 10% NP-40 and Complete Mini Protease Inhibitor Cocktail tablets (Roche, www.roche-applied-science.com). All embryonic extracts were prepared from 3- to 18-h-old embryos. Crude embryo extract was prepared in NET buffer supplemented with 10% NP-40 and Complete Mini Protease Inhibitor Cocktail tablets (Roche). Embryo nuclear extract was prepared as described previously [39]. Co-immunoprecipitations and Western blot analysis were carried out according to standard protocols, using the conditions described previously [25].

### D. melanogaster strains, isolation of *spf45* mutant alleles, and construction of the *spf45* rescue construct.

Descriptions of marker mutations and balancers not listed below or in the text are described on Flybase (http://www.flybase.org). For the genetic interaction assays, the following mutations were used: *Sxl^f1^* ^,^ *, Sxl^7BO^* [40], *rad201^1^* (described on http://www.flybase.org), and *rad201^ZII670^* [31] *spf45^J23^* were generated by imprecise excision of the *P-element* KG2503 [27]. Because of the low frequency of transposase-induced excision events we also screened for chemical-induced excision events by feeding males carrying the KG2503 *P-element* 0.007 M DEB for 24 h, as described previously [41]. Only one derivative, *Df(2Lh)D1,* proved to be deleted for the *spf45* coding sequence. Lethal mutations that failed to complement *Df(2Lh)D1* were isolated in a standard EMS (0.025 M) mutagenesis screen. The *P[spf45+]* genomic rescue construct was made from a 3.1-kb genomic region generated by PCR using the primers ATGGTTCGGCTAAACAGACG and CGTTTTGACTTCGACCGATT and TA-cloned into the pCR4-TOPO vector (Invitrogen, www.invitrogen.com). The resulting *PstI-NotI* fragment was inserted into the pCaSpeR4 transformation vector [42]. Germline transformants were obtained by standard methods and each transgenic line was tested for its ability to rescue the different *spf45* mutant phenotypes. The data presented in this paper are obtained with transgenic line 16.

### RT–PCR analysis.

RNA was isolated from either adults or embryos using standard methods. For analysis of the *Sxl* reporter construct, the first-strand synthesis was carried out using the Superscript First-Strand Synthesis System (Invitrogen). The conditions and primers used to amplify the *Sxl* reporter construct have been previously described [30] and were carried out with the following modifications: the PCR reactions were performed in a 25 μl volume with 2 μl of the RT reaction with the Z1 lacZ primer and the *Sxl* primer using the Expand High Fidelity PCR System (Roche). The PCR conditions were as follows: 95 °C for 3 min followed by 10 cycles of 95 °C for 45 s, 62 °C for 2 min, and 68 °C for 45 s. This was followed by 30 cycles of 95 °C for 45 s, 62 °C for 2 min, and 68 °C for 2min30s, and a single final step at 68 °C for 7 min. A 1% aliquot of the first PCR reaction was amplified further in a 25 μl volume using the Z2 lacZ primer and the *Sxl* primer. The PCR conditions were as follows: 94 °C for 10 min followed by 25 cycles of 95 °C for 45 s, 61 °C for 2 min, and 72 °C for 1min 30s, and a single final step at 68 °C for 5 min. Products were detected on a 2% agarose gel by staining with ethidium bromide.

For analysis of the *rad201/CG2412* splicing products, the first-strand synthesis was carried out with the following gene-specific primers: ATATCAGCACCAGTGGCAGCC for the product spanning intron 1, or TTAGACCACGCCTGCATCCGT for the product spanning intron 2, at 55 °C for 30 min using Superscript III (Invitrogen) added directly to the cocktail used for the Roche Hi-Fidelity Expand kit (5 ml buffer, 2 ml 10 mM dNTPs, 0.5 ml RNAseOut [Invitrogen], 0.75 ml Taq polymerase in a 50-ml reaction). Following a 10-min heat-inactivation step at 95 °C, the PCR reaction was initiated with the following primers: CCATGGACCTCAGGCCTATGATT for the product spanning intron 1, or GGCTGCCACTGGTGCTGATAT, for the product spanning intron 2. The PCR conditions used for intron 1 were as follows: 94 °C for 10 min followed by 30 cycles of 95 °C for 1 min, 59 °C for 2 min, and 72 °C for 1 min, followed by a single final step at 72 °C for 10 min. For intron 2 the conditions were as follows: 94 °C for 10 min followed by 30 cycles of 95 °C for 1 min, 55 °C for 2 min, and 72 °C for 1 min, followed by a single final step at 72 °C for 10 min.

### Yeast two-hybrid analysis.

Yeast two-hybrid assays were performed using standard methods. Full-length cDNAs or derivatives were cloned in-frame into either the pACT2 vector (Clontech, www.clontech.com) for expression of a GAL4 DNA–activation domain fusion protein or into the pAS2–1 vector (Clontech) for expression of a GAL4 DNA–binding domain fusion protein. The two plasmids were cotransformed into the diploid strain, pJ69A/SL3004, and plated onto selective media with 1 mM 3-amino-1,2,4-triazole added (Sigma, www.**s**igmaaldrich.com) to assay for GAL4-dependent transcription of the HIS3 reporter construct.

### Rescue of *E. coli recG* mutant.

*spf45* and *spf45^J23^* cDNAs were cloned in-frame into the expression vector, pGEX (Amersham, http://www4.amershambiosciences.com), along with the negative control, pGEX-2T. Each construct was transformed into the *recG* mutant strain, N4256 [43]. After induction with IPTG, the strains were plated on Luria-Bertani plates containing either 0.05% or 0.075% MMS. Plates were incubated overnight at 37 °C, and the number of colonies scored.

### MMS sensitivity assay.

Larval sensitivity to MMS treatment was measured as follows: parent flies were allowed to lay eggs in a vial for 24 h. The resulting progeny were treated with MMS during the larval stage (48–72 h after egg laying was initiated) by layering 250 μl of a 0.075% MMS solution onto the media. Survival was judged by the number of the experimental animals that survived to adulthood compared with their control siblings.

### *P{w^a^}* assay for double-strand break repair.

The *P{w^a^}* transgene and a detailed description of the assay have been described previously [32,44]. Briefly, *P{w^a^}/Y; Df(2Lh)D1/spf45^J23^; TMS,Sb {D2–3}/+* males were mated to *y w P{w^a^}* females and the eye color of all female *Sb^+^* progeny scored. For the controls, *P{w^a^}/Y; Df(2Lh)D1/Cy0; TMS,Sb {D2–3}/+* males were mated to *y w P{w^a^}* females and the eye color of all female *Sb^+^* progeny scored.

### Meiotic nondisjunction assay.

To measure the frequency of X–X nondisjunction, homozygous *w/w; spf45^J23^* females were crossed to *y, w^+^ / B^s^Yy+* males and the progeny scored. In the female, meiotic nondisjunction will generate null-X eggs and XX eggs. Null-X eggs will support development if fertilized with a paternal X chromosome, leading to a *y, w^+^* XO male. Similarly, XX eggs will support development if fertilized by the Y chromosome, leading to a XXY (*w; B^s^*) female. The number of exceptional progeny recovered gives a rough indication of the effect of *spf45* on the frequency of X–X nondisjunction.

### *rad201* (CG2412) gene structure and open reading frame.

To determine the gene structure of CG2412, cDNAs were amplified by RT–PCR from total RNA isolated from embryos and the intron/exon junction deduced by comparison with the published genomic sequence. This analysis revealed a discrepancy between our placement of the intron/exon boundaries and the electronically annotated gene structure of the Berkeley *Drosophila* Genome Project (Annotation Release Version 4.3). Our data indicate that the open reading frame begins in exon 1 (sequence coordinates: 4,677,548–4,677,798), continues through exon 2 (4,677,858–4,678,372), and ends in exon 3 (4,678,435–4,678,679). This open reading frame encodes a 248–amino acid protein that has increased sequence homology with predicted proteins in the mosquito, *D. pseudoobsura,* and human genomes.

### GST pull-down assays.

Full-length cDNAs for *rad201* (CG2412) and U2A′ were cloned into pGEX (Amersham), and the resulting fusion proteins were expressed and purified from E. coli using standard methods. For the pull-downs, 20 μl of glutathione-immobilized beads (Pierce, http://www.piercenet.com) were incubated with 60 μg GST or 80 μg of GST-tagged protein for 1 h at 4 °C, before adding 2.5 mg of protein extract (approximately 100 μl) made from 3- to 18-h-old embryos. The bead-and-extract mixture was incubated overnight at 4 °C, then washed and eluted with 22 μl of 2X SDS loading buffer. Western blots were performed using antibodies against SNF or SPF45, as described above. For experiments in which the extracts were treated with RNase, 1/10 volume of RNase A (10 mg/ml) and 1/20 volume RNase T1 (100,000 units/ml) were added to the crude extract.

## Supporting Information

### Accession Numbers

Accession numbers used in this paper from the US National Center for Biotechnology Information (http://www.ncbi.nlm.nih.gov) database are: D. melanogaster (NP_001015406), humans (NP_116294), and the DRT111 protein from A. thaliana (NP_174336).

## Abbreviations

RRM: RNA-recognition motif
